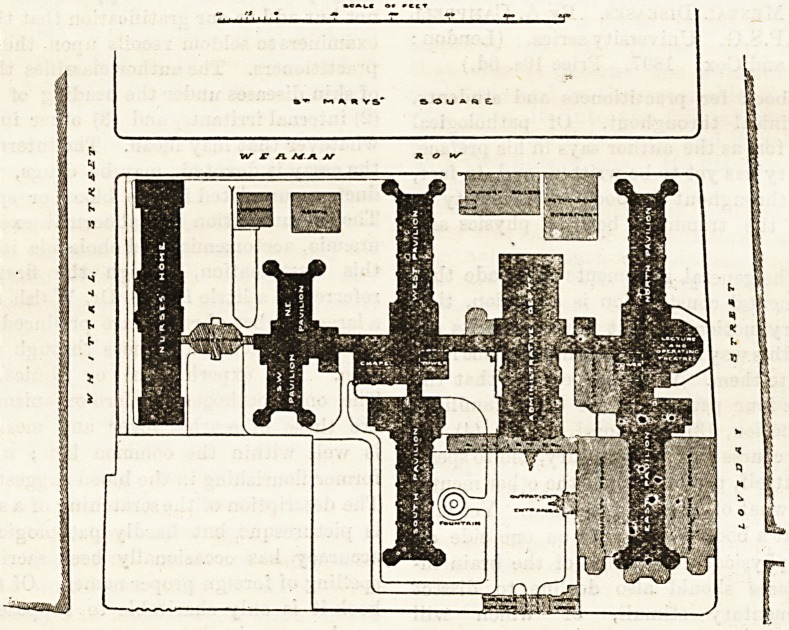# Hospital Construction

**Published:** 1897-07-17

**Authors:** 


### The Institutional Workshop.

### HOSPITAL CONSTRUCTION.

### OPENING OF THE NEW BIRMINGHAM GENERAL HOSPITAL.

With great ceremony, in the presence of a large concourse of people, and amid the cheers of an immense multitude, the New Birmingham General Hospital was opened on July 7th by H.R.H. The Princess Christian. It was a day which no doubt will long be remembered in Birmingham as marking the completion of a great undertaking, and the launching of the new hospital on what we hope will be a prosperous career. It would, indeed, have been a reproach to a city so progressive as Birmingham has shown herself to be, a city which in the past thirty years has become altered out of all recognition, if amid all the changes and improvements manifest on every side the hospital had been allowed to remain on the old unsuitable site, amid its crowded and insanitary surroundings, and in a building which was the despair of all who had tried to improve it. Not that the governors had been careless or indifferent. No one who recognised the difficulties and dangers amid which the treatment of disease was undertaken in it during the early sixties could remain indifferent to the state of affairs then existing. Money was poured out with lavish hands, upwards of £50,000 having been spent in improving and extending the old buildings, but the evils were inherent and ineradicable, and the people of Birmingham and the district, while rejoicing in the possession of such a perfect hospital as the one just opened, ought not to forget what they owe to that small band of earnest and far-seeing men who had the boldness to propose and to urge that all the good money that had been spent in the old place should be thrown away rather than that the evils inherent in it should be perpetuated, and had the inspiration to lead liberally in the subscriptions which made it possible to erect a new and perfect hospital upon a new and vastly better site.

In describing the new hospital we must first give expression to the admiration which we felt, and which we think everyone will feel who sees it, at the noble proportions and handsome character of the building. In their fresh newness the walls perhaps display a certain monotony of colour, which, however, will soon wear off; but in regard to form we have nothing but praise to offer. The free working of the terra-cotta facings and the really beautiful distribution of light and shade by means of the balconies, the cloister arches, and the deep set windows of the turrets, give to the building a richness of surface such as is rarely seen, while for picturesqueness of outline it is all that could be desired.

The general scheme of the hospital will be understood on reference to the accompanying plan.

On entering the administration block, we find ourselves among the rooms allotted to the resident medical officers. We then come to the main corridor running right and left to the extreme ends of the building; to the right to a double three-storied pavilion, the lower floor of the front half of which is occupied by a portion of the out-patient department; to the left to a similar double three-storied pavilion, the lowe floor of the front half of which is occupied by the board-room and committee - rooms; beyond that to the left again to a double pavilion of eight-bedded wards, with several single-bedded wards attached; and still further to the left, again, ending in a conservatory which serves at the same time as a disconnection and a means of union between the hospital and the nurses' home, which lies beyond. At the back of one portion of the building are the boilers with a large chimney, and behind the other part are two entirely detached buildings—one for septic cases, and the other for the isolation of any cases of infectious disease which may be accidentally admitted or may arise in the wards.

Returning again to the administration block, where we first entered, it is to be noted that the portion at the further side of the main corridor is occupied chiefly by dining-rooms—one for the officers, one for the nurses, and one for the servants. Above these are two storeys of servants' bed-rooms, and at the top of the whole block are the kitchen, various stores, and the servants' sitting-rooms.

The hospital provides ten large wards of 24 beds, and six smaller wards for 12 beds each, together with a number of single-bed wards, some of which are in the septic and infectious departments. Altogether arrangements are made for 346 patients; in addition to which there is ample accommodation for the staff and for servants, and a separate nurses' home with sitting and separate bed rooms for over 100 nurses. The whole of the building is fireproof.

The out-patients' waiting-hall is a very pretty room, the walls being of a delicately-tinted faïence ware, with a semi-glazed surface, above a richly-glazed majolica dado supplied by Messrs. Campbell and Co., of Stoke. The proportions of the room, moreover, are good, the columns which carry the walls of the wards above are very handsome, and the clerestory windows by which the room is lighted give to it an air of brightness very different from what is commonly seen in such places.

The two gems in the building are, however, the main operating theatre and the chapel. There are three operating theatres in the hospital, the main one being situated over the large lecture theatre. It is semi-circular in form, the curved side and a considerable portion of the roof being filled with windows. So far as daylight is concerned, it is practically in the open air. The floor is of marble mosaic, the walls are of marble and alabaster—specially treated so as to be non-absorbent—the ceiling is of Birmingham waterproof cement painted, and the gallery for the students is a skeleton arrangement built of steel tubing, with planks of teak tier above tier. It is to be noted that the students face the windows—not altogether an ideal position. In the lecture theatre below, the seats are, of course, arranged the other way.

The chapel is very beautiful. The walls are lined internally with marble and alabaster, a special gift from Mrs. J. C. Holder; the west windows and the pulpit are a memorial to the late Dr. Bartlett, one of the side windows is in memory of the late Dr. Jolly, and Mrs. J. B. Clarke has given the oak seating and the fittings. On entering this chapel, one cannot but be struck by the restful character which is given to it by the absence of an east window. The walls are rich and warm in tone, the light is plentiful, but there is nothing to dazzle the eyes of the worshippers or prevent them seeing the preacher.

The wards are very simple. The larger ones contain 24 beds, with a window for each, eight feet of wall space per bed being allowed. A day-room and a couple of small wards are attached to most of the wards. The sanitary arrangements are in towers at the end of the wards, and in the case of the pavilions, which point southwards, an open verandah, accessible from the ward, fills up the space between the towers. The walls of the wards are throughout of adamant plaster, a dado four feet six inches high being enamel painted, and the wall above being distempered.

The flooring is of teak boards (not blocks) laid upon a bituminous bed and edge nailed direct into the underlying breeze concrete. This concrete lies on adamant blocks supported by rolled steel joisting. Thus the whole thickness of the floor is solid, but Mr. Henman is of opinion that the varying densities of the materials used in juxtaposition will more or less neutralise sound waves. As a fact, we noticed how little noise was caused by walking on these floors. All the corners between walls and floors and ceilings are rounded off.

If space allowed we might fill pages in describing the many devices met with on every side which testify to the careful thought given to the matter by Mr. Henman, the architect, and by Mr. J. C. Holden, the chairman of the hospital. No one could witness the spontaneous unanimity with which the vast assembly in the pavilion rose to their feet on the entrance of Mr. and Mrs. Holder, and the cordiality with which they welcomed them as they proceeded to the daïs, without recognising that the people of Birmingham were not unmindful of the great things which Mr. Holder had done for their hospital, both by his generous gifts and by his personal attention to its interests; and when we had the pleasure of being shown over the building by him afterwards we could not but feel the influence of his enthusiasm, and see that he had been the moving spirit in the introduction of much that is most novel and important in its construction. We now come to the great peculiarity of the building, the matter which above all others renders it of interest to those engaged in similar undertakings, viz., the great experiment which is being made in regard to ventilation. The plenum system of ventilation has been many times tried, but nowhere in England has any attempt been made to apply it on so large a scale to the ventilation of a hospital. What has to be understood is that what is done at the New Birmingham General Hospital is not a mere matter of driving air into the wards, but is a complete and consistent perflation of the whole building by properly-warmed air driven by mechanical power. We use the word perflation because the air is not merely blown in and allowed to filter out, but is blown right through, and remains under pressure until it escapes at the outlets arranged for it.

To attain this object the whole building is made as air-tight as possible; every window is permanently closed, not a crevice is allowed by which the air can escape, and except in a few of the officers' rooms there is not a chimney or a fireplace in the institution. Air is drawn in by a fan, being cleansed on its way by passing through a wet screen; it is then warmed when warming is required, and is driven onwards by the power of the fan through wide passages, carried mostly in the thickness of the walls, into the different parts of the building. These air-passages terminate in many cases at a window-sill, the opening being the full width of the window, and being protected by a sheet of glass extending upwards about a couple of feet, by means of which the air is made to enter with an upward momentum. Another mode of termination of the air-passages is by means of large openings in the upper parts of the rooms. But in all cases the air has to leave the rooms near the floor; it is then carried by air-shafts built in the walls to turrets above the roof, protected by flaps and louvres so that it may escape into the open uninfluenced by movement of the outer atmosphere. All the time, and at every point in the hospital up to the moment of escape from the turrets, the air is under a certain slight pressure, sufficient to make it always tend to flow outwards.

It is to be remembered that the whole of the air entering the building is to be raised to a uniform temperature of between 60 deg. and 62 deg. Fahr., with the option of raising it in certain rooms to 70 deg. if necessary. This being the case, it is considered unnecessary to provide any further means of heating the building there not being a fireplace nor a steam-pipe in either wards or passages.

At four selected points in the basement air is drawn in through moistened screens, by which it is cleansed of suspended impurities and brought to a state of humidity suited to the temperature of the interior. These screens are warmed by steam-pipes placed outside, otherwise they would freeze solid in cold weather. The air is then passed over large batteries of steam-pipes, and propelled by rotatory fans driven by electric motors. The air-ducts are large, roomy passages, from which flues lead up to the various wards, rooms, and passages, and in them are water-pipes and connections for hose-pipes, so that the whole may be properly flushed and cleaned. At the base of each flue are additional steam-coils, with regulating valves, so that the temperature and air supply to each apartment may be adjusted to requirements. Eight fans and electro-motors are employed, being arranged in couples so as to prevent stoppage of the ventilation in case of accident or repair. The total power developed by the whole of the motors is said to amount to about 55-horse power, about three-quarters of which is required. The power is provided by the Electric Supply Company at a phenomenally low rate in consideration of its being continuously used. The size of the fans is so calculated as to provide for changing the whole of the air throughout the buildings from seven to ten times per hour. The whole of the ventilating and heating arrangements have been engineered by Mr. William Key, of Glasgow.

Now, in considering the probable efficiency of these ventilating appliances, we must first state that on the day of opening they worked perfectly well. The whole of the buildings appeared to be supplied, so far as the senses could tell, with an ample quantity of fresh air, and by repeated and careful examination in the neighbourhood of the various orifices we were able to assure ourselves that there was no perceptible draught, even very close to where the air was found to be pouring in. Perhaps as good a test as any of the effectiveness of the system was that furnished by the wards in which tea was provided for the very numerous guests. These rooms were filled with people, yet they were quite fresh.

But, in fact, we have no doubt that the thing can be done, and that it is possible by mechanical means to drive an enormous amount of air through a building. The question is — Will it be done? (*a*) Will the managers go on day and night, from year's end to year's end, supplying the full quantity of air? and if in this individual instance they do so (*b*) will it be an economical proceeding to pay so much for a commodity which can be had for nothing by the simple process of opening the windows? To these we would add two other questions. (*c*) Will the air supplied continue to be clean and sweet when these air ducts have been in use a few years? and what will happen supposing the fans to fail, either from accident or from stoppage of the electric supply?

In regard to question (*a*) we would point out that whatever the cost the managers of the Birmingham Hospital are bound to go on pumping in the air required. They have no alternative. We give all due praise to Mr. Henman for having constructed a beautiful and most excellent hospital, but our praise is entirely conditional on the loyalty of the committee in carrying on the scheme of mechanical ventilation which he has provided, for it is clear enough that if natural ventilation were to be depended on the plan of the building would be far from perfect. Except for the promise of thorough artificial ventilation we could not possibly approve of the planning of the ground floor of the new hospital. The main corridor has but very meagre cross ventilation, and there is no attempt at separating the wards from it except in the case of those to the south. All the rest of them open direct on to the corridor, and under a natural system of ventilation there would be complete community of atmosphere throughout the building, the very thing that was the curse of the old place. Nor could we approve of placing a large out-patient department directly under a block of wards, nor the placing of the gynæcological wards so near to the burn wards, except on the assurance that air is to be driven into and driven out of each ward without any possibility of obtaining access to any other part of the building.

As to the next question (*b*), we gather from the architect that the additional cost to the building entailed by the mechanical ventilation has not been more than between £2,000 and £3,000; and in regard to the current expense, he considers that the economy of producing heat by a central properly constructed furnace, instead of by a series of wasteful open fires, and the saving of dirt and of labour by not having to carry coal to the wards, will repay the expense of running the fans. But outside and beyond all this, he maintains that even if it does cost money the hospital will be well repaid by the cleanliness of the air supplied, and by the certainty with which it will be driven through every part of the building in entire independence of the temperature of the air outside.

As to (*c*) the maintenance of the air ducts in a state of cleanliness, we are assured that provision is made for washing them throughout. Lastly, as to the chance of breakdown, it is pointed out that the fans and motors are run in pairs so as to reduce the danger to a minimum; but what is to be done if the electric supply should fail we are not told, and we are afraid that to that question no satisfactory answer can be given. We prefer then still to speak of this hospital as an experiment, one which is most important, and the results of which all those who are interested in hospitals will watch with the greatest interest. We hope in a few months' time to be able to publish figures giving the actual cost. In the meantime we must give all praise to the care and thought which has been bestowed on every part of the building, and on the consistency with which every detail has been worked out and made to harmonise with the proposed method of ventilation. If, after all this, the system should fail, mechanical ventilation will be utterly condemned; if, on the other hand, it should succeed, we cannot but see that many new possibilities will be opened up in the construction of town hospitals; and great will be the credit due to the pioneers.

## Figures and Tables

**Figure f1:**